# Registration Law of Kentucky

**Published:** 1852-03

**Authors:** 


### REGISTRAVION LAW OF KENTUCKY.

We are very happy to be able to announce the enactment of the following law by the late Legislature of Kentucky. Its adoption will form a new era in the medical history of the State.

### AN ACT TO PROVIDE FOR THE REGISTRATION OF BIRTHS, DEATHS AND MARRIAGES IN KENTUCKY.

Sec. 1. *Be it enacted by the General Assembly of the Commonwealth of Kentucky,* That it shall be the duty of all clergymen, or other persons, who shall hereafter celebrate or perform the marriage ceremony within this Commonweath, to keep a registry of all marriages celebrated by them, showing the names, ages, residence, and place of birth of the persons married, whether they were single or widowed, and the time of the marriage.

Sec. 2. It shall be the duty of all physicians, surgeons, and midwives to keep a registry of all births and deaths at which they have professionally attended, showing, in cases of birth, the time and place of birth, name of the father, and maiden name of the mother, and their residence, sex and color of the child, together with its name, if it shall receive one, and whether it was born alive or dead; and showing, in cases of death, the time, place, and cause of death, the name, age, sex, color, and condition, (whether single, married, or widowed,) name and surname of parents, occupation, residence, and place of birth of the deceased: *Provided,* that in case of a birth of a slave, the name of the owner shall be given in place of the names of the parents, and, in case of the death of a slave the owner's name may be given in place of the condition, occupation, and residence: *And, provided further,* that when two or more physicians, surgeons, or midwives may have attended professionally at any birth or death, that physician, surgeon, or midwife who is oldest in attendance shall make the registry.

Sec. 3. It shall be the duty of clergymen, physicians, &c., above named, to deposit in the county clerk's office of the counties in which such births, &c., occur, on or before the 10th day of January, in every year, the said registry, or a copy thereof, embracing the period of one year, ending on the 31st day of December last preceding the time of deposit; and the clerk shall deliver copies of the same to the assessor.

Sec. 4. It shall be the duty of the assessors, while making their lists of taxable property, to ascertain and record, in a list separate from the list of taxable property, all the births, marriages, and deaths which shall have occurred within their respective counties in the twelve months ending on the 31st day of December last preceding the time of assessment. with all the items of time, place, &c., herein directed to be inserted in the registries above named; and they shall make strict inquiry of all heads of families, and shall use the registries of clergymen, &c., above named, in order to obtain correctly the information herein required. They shall return said lists of births, &c., with the registries of clergymen, &c., aforesaid, to the clerks of the county courts at the same time they return their lists of taxable property; and the clerks shall copy said lists of births, &c., and transmit the copies to the auditor of public accounts with the lists of taxable property. The clerks shall be paid at the same rates they are paid for copying the list of taxable property. The assessor shall be allowed two cents for each birth, marriage, or death recorded as herein directed, to be paid in the same manner as for making the lists of taxable property: *Provided*, that it shall be lawful for any assessor to record, separately, the time, place, &c., of any birth, marriage, or death which may have occurred prior to the time which the list then being made embraces, or which may have occurred without this Commonwealth; for every entry so made, the party causing it to be done shall pay the assessor two cents.

Sec. 5. It shall be the duty of the auditor to make, from all the lists of births, marriages, and deaths so transmitted to him, tabular statements showing, in a condensed form, the information herein required to be preserved, keeping the statistics of each county separate: and to cause five hundred copies of the same to be printed in pamphlet form, on or before the first day of January in every year; to transmit not more than five nor less than two copies to each county court clerk's office in this Commonwealth, one of which shall be forever carefully kept in such office, and the remainder distributed for the use of the citizens of their respective counties. He shall cause to be printed suitable blanks for the use of assessors, clergymen, physicians, &c., with separate columns for each of the items of information herein required, and send a sufficient number of said blanks to the clerks of each county court for distribution. He shall annex to said blanks such instructions as he may deem necessary to secure the faithful execution of this act.

Sec. 6. To enable the assessors to obtain full and correct information touching the facts herein required to be ascertained, they shall have full power to swear and interrogate any person in thetr respective counties for that purpose; and it shall be the duty of all such persons, when thereto required by the assessor, with or without oath, to give him, fully and truly, all the information he or she may possess touching any of said facts.

Sec. 7. The several county court clerks shall forever carefully preserve the lists of births, &c., and the registries of clergymen, &c., herein required to be returned to them, for the use of the public.

Sec. 8. The said lists of births, marriages, and deaths, returned to the clerks of the county courts by the assessors, as also the original tabular record herein required to be made and kept by the auditor, or a duly certified copy of any birth, marriage, or death from either of them, given and certified by the keeper of such records, shall hereafter be admitted and received, in all courts in this Commonwealth, as *prima facie* evidence of any such birth, marriage, or death therein recorded or so certified.

Sec. 9. Any person failing to discharge and perform any of the acts or duties herein imposed and required to be done, shall, for every such failure, be fined in a sum not less than five nor more than twenty dollars, to be reeovered by warrant before a justice of the peace, or by presentment by the grand jury.

